# The glutamate release inhibitor riluzole increases DNA damage and enhances cytotoxicity in human glioma cells, *in vitro* and *in vivo*

**DOI:** 10.18632/oncotarget.26854

**Published:** 2019-04-19

**Authors:** Atif J. Khan, Stephanie LaCava, Monal Mehta, Devora Schiff, Aditya Thandoni, Sachin Jhawar, Shabbar Danish, Bruce G. Haffty, Suzie Chen

**Affiliations:** ^1^ Department of Radiation Oncology, Memorial Sloan Kettering Cancer Center, New York, New York 10065, USA; ^2^ Department of Radiation Oncology, Rutgers-Robert Wood Johnson Medical School, Piscataway, New Jersey 08854, USA; ^3^ Susan Lehman Cullman Laboratory for Cancer Research, Department of Chemical Biology, Ernest Mario School of Pharmacy, Rutgers University, Piscataway, New Jersey, USA; ^4^ Department of Surgery, Division of Neurosurgery, Rutgers-Robert Wood Johnson Medical School, Piscataway, New Jersey 08854, USA

**Keywords:** glutamate, glioma, riluzole, radiation therapy

## Abstract

**Purpose:**

High-grade gliomas are lethal malignancies that cause morbidity and mortality due to local progression rather than metastatic spread. Our group has previously demonstrated that human GRM1 (hGRM1) is ectopically expressed in melanocytes leading to a transformed phenotype. Riluzole, a glutamate release inhibitor, leads to apoptotic cell death via DNA damage. Recent work has demonstrated the pathological significance of the related mGluR3/GRM3 (protein or gene: hGRM3) in gliomas. We evaluated the effect of riluzole on glioma cells.

**Experimental Design:**

Western blot analysis and immunofluorescence was performed to assess for GRM3 expression in commercially available and patient-derived glioma cells and for functional analysis of GRM3 using receptor agonist/antagonists and downstream effectors, ERK and AKT phosphorylation, as the read-out. Glutamate secretion by glioma cells was measured using ELISA. Flank and intracranial mouse xenograft models were used to assess growth delay with the glutamate release inhibitor, riluzole (RIL). Immunofluorescence was used to evaluate 53BP1 or γ-H2AX foci after RIL.

**Results:**

GRM3 was expressed in most tested glioma samples, and strongly expressed in some. Glioma cells were found to secrete glutamate in the extracellular space and to respond to receptor stimulation by activating downstream ERK. This signaling was abrogated by pretreatment with RIL. Treatment with RIL caused an increase in DNA damage markers, and an increase in cellular cytotoxicity *in vitro* and *in vivo*.

**Conclusions:**

We have demonstrated that pretreatment with the glutamate-release inhibitor riluzole sensitizes glioma cells to radiation and leads to greater cytotoxicity; these results have clinical implications for patients with glioblastoma.

## INTRODUCTION

High-grade gliomas are particularly vexing tumors that indiscriminately devastate both adults and children alike. The addition of concurrent and post-concurrent temozolamide to radiation therapy (RT) has been a significant advance in glioblastoma therapy [1]. Still, the overall prognosis remains decidedly poor, even in patients with silenced O^6^-methylguanine-DNA methyltransferase (MGMT) expression. While patients with silenced MGMT benefit from temozolamide therapy, those with MGMT active tumors do not typically benefit with temozolamide. As such, the search for alternate therapies for these patients is urgent. Furthermore, the majority of glioblastoma recurrences occur in and around the site of the index lesion (with little propensity for distant disease). As such, novel agents should ideally be deliverable with and enhance the effects of cranial partial brain RT.

Glutamate abnormalities have been extensively described in preclinical work in gliomas. Prolonged synaptic activation from excess glutamate can lead to “excitotoxic” cell death, a process described decades ago [2] and now known to be a final common death pathway in many neurological injuries [3, 4]. The excitatory amino acid transporters 1 and 2 (EATT1 and 2) are Na^2+^ dependent glutamate transporters expressed on normal astrocytes that maintain extracellular glutamate levels at uM concentrations by uptaking glutamate [5]. Glioma cells lack expression of EATT2 and appear to have EATT1 mislocalized to the nucleus, resulting in no functional glutamate uptake [6]. de Groot et al., demonstrated lower levels of EATT2 expression in high-grade gliomas and no detectable EATT2 in glioblastomas [7]. At the interface of glioma and normal brain, IHC analysis has demonstrated low EATT2 levels on the tumor side and prominent expression in astrocytes in uninvolved brain [8].

Paradoxically, glioma cells *release* glutamate through system X^c^, a glutamate-cystine antiporter system composed of two proteins, xCT and CD98 [6]. Savaskan *et al.*, have conclusively demonstrated that xCT levels are elevated in gliomas, xCT ablation by siRNA results in massive reduction in glutamate secretion, and that conditioned media from glioma cells results in neuronal degeneration only if xCT is functional [9]. System X^c^ is also a key determinant of intracellular glutathione reserve (by enriching cellular levels of cystine), thus conferring an additional survival advantage to glioma cells. In summary, decreased uptake and excess secretion of extracellular glutamate in the microenvironment facilitates intracranial glioma progression and expansion.

Glutamate also acts as a trophic factor for glioma cells. Ciceroni *et al.*, have demonstrated that glioma-initiating CD133-positive stem cells express the metabotropic glutamate receptor 3 (GRM3/mGluR3) and that pharmacologic blockade of mGluR3 results in committed differentiation into benign astrocyte-like cells. In addition, activation of mGluR3 inhibits bone-morphogenetic protein receptor induced differentiation by activating the MAPK pathway, and that treatment with an mGluR3 receptor antagonist suppresses *in vivo* tumor growth in a mouse intracranial glioblastoma model of implanted CD133^+^ stem cells (or tumor-initiating cells as we refer to them) [10].

Our earlier work led to the identification and confirmation that ectopic expression of a murine neuronal receptor; metabotropic glutamate receptor 1 (mGRM1) in melanocytes was sufficient to induce spontaneous melanoma development *in vivo* [11–13]. We further demonstrated that GRM1 expression resulted in signaling through the MAPK and PI3K pathways, promoting growth and invasion, and that treatment with riluzole (a glutamate release inhibitor) resulted in DNA damage, apoptosis and cell death. Our discovery that riluzole induces DNA damage, likely mediated by a reduction in glutathione levels within the transformed cells, opens up possibilities of combining riluzole with agents such as ionizing radiation that increases sensitivity in cells with damaged DNA.

Riluzole is an FDA approved drug for the treatment of amyotrophic lateral sclerosis (ALS) and has off-label uses in other psychiatric and neurologic disorders. Riluzole possess both glutamatergic modulating and neuroprotective properties, although the precise mechanisms have not been fully delineated [14–16]. Because riluzole crosses the blood brain barrier, it is of particular clinical relevance since candidate drugs with documented CNS penetration are relatively uncommon.

In the current communication, we examined the potential for enhanced cytotoxic effects with the addition of ionizing radiation to riluzole in human glioma cell lines. We hypothesize that riluzole will be a radiation sensitizer for the treatment of high-grade glioma.

## RESULTS

### GRM3 is expressed in glioma cells

**Figure 1:**
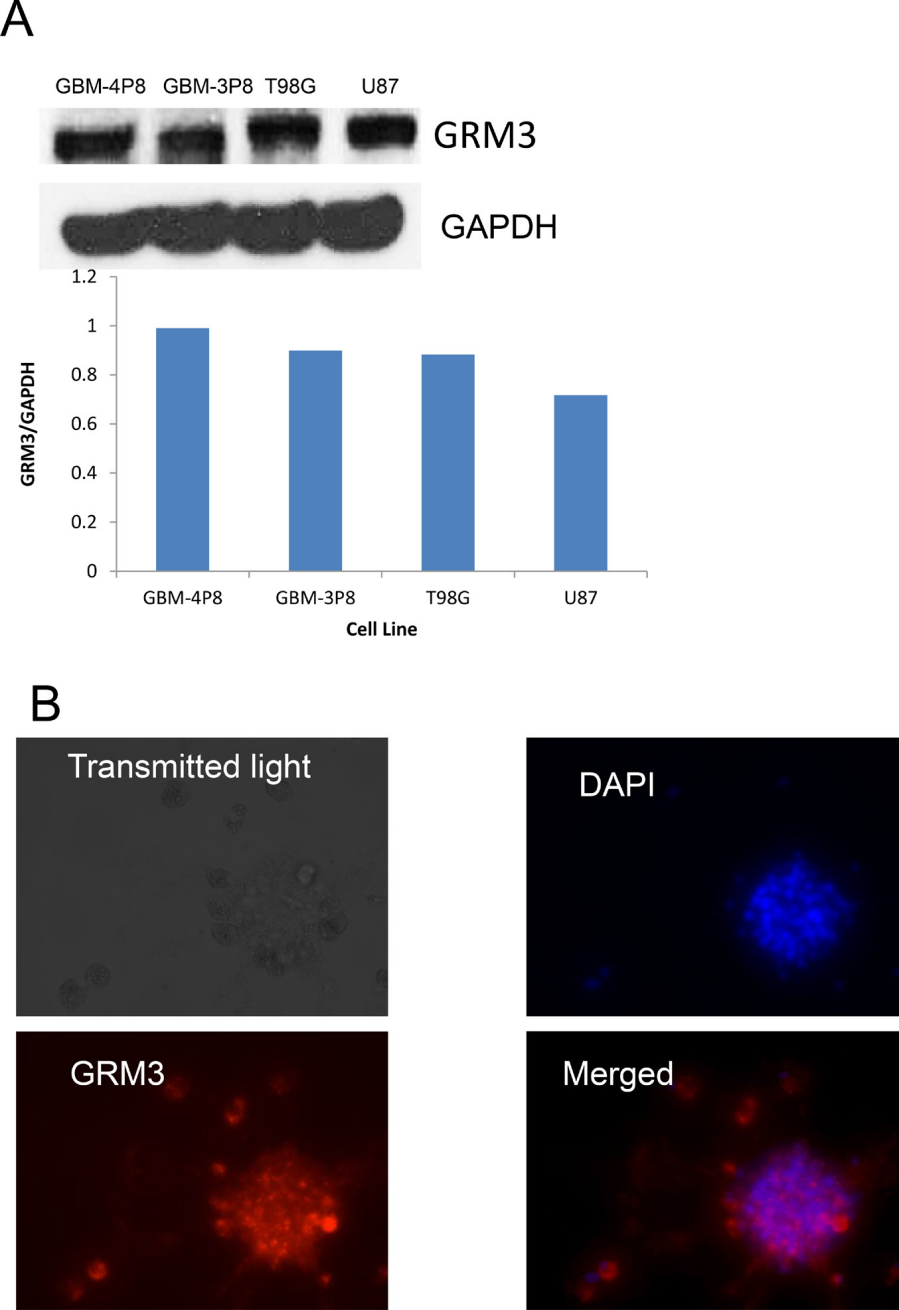
GRM3 is expressed in human gliomas (**A**) Western immunoblots of commercially available human glioma cell lines (T98G and U87) and two primary patient-derived cell lines (GBM 3P8 and GBM 4P8). The same blot was probed with GAPDH to show equal loading. (**B**) Immunofluorescence demonstration of DAPI, rhodamine-GRM3 and merged of GRM3 and DAPI in an example of primary patient derived cells.

We wanted to confirm that GRM3 was expressed in human glioma cells. On western blotting, we were able to demonstrate GRM3 expression in both commercially available cell lines (U87 and T98G) and in our primary patient-derived cells. In our cohort of primary samples, 8/12 samples had detectable expression of GRM3 by western blot, of which the strongest expressers (GBM-4P8 and GBM-3P8) are shown (Figure 1A). We were also successful in demonstrating expression of GRM3 using immunofluorescence (IF) in our primary samples, an example is shown in Figure 1B and in Supplementary Figure 1.

### Glioma cells secrete glutamate into the extracellular microenvironment

**Figure 2:**
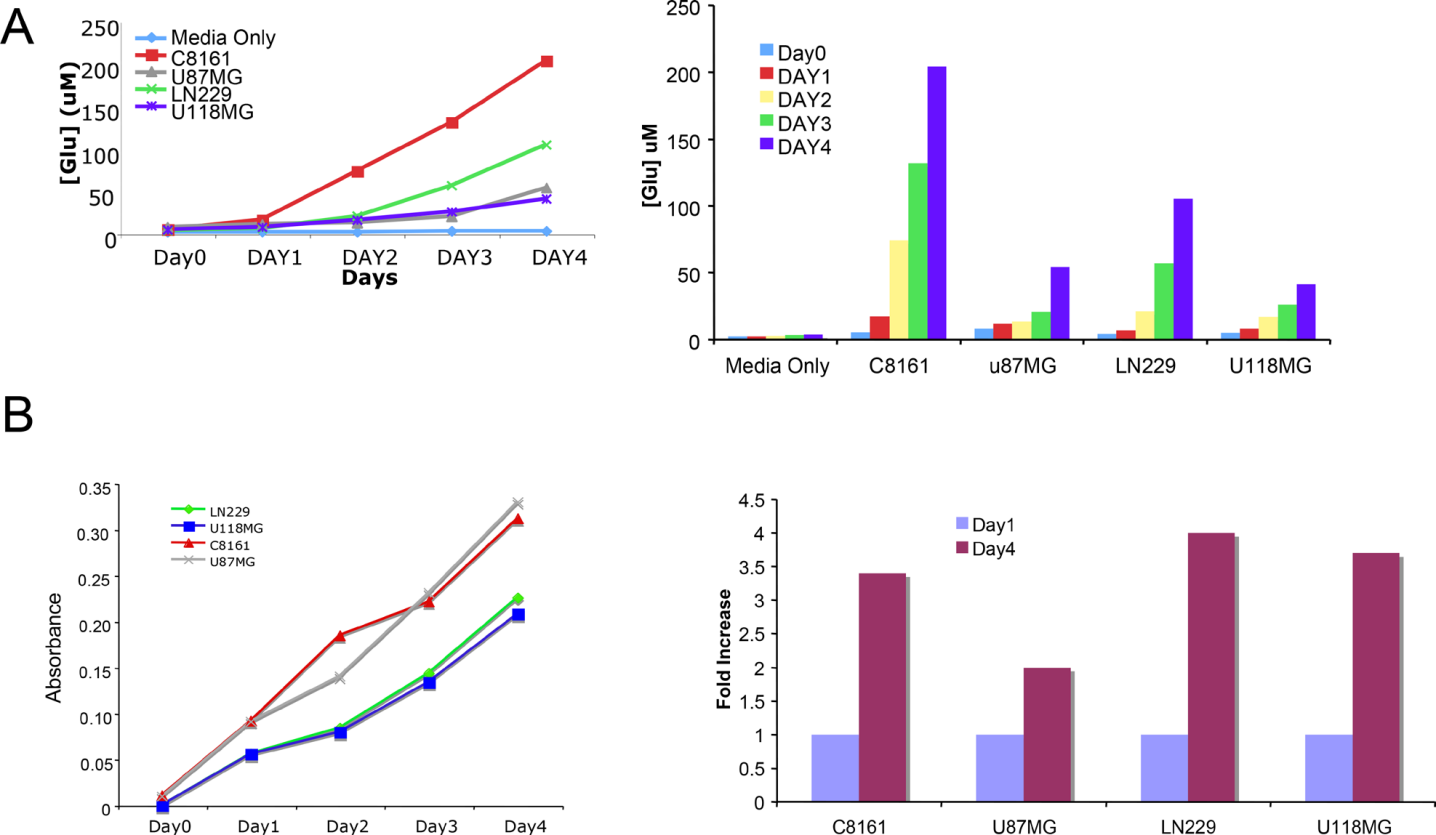
Glutamate is secreted into the microenvironment by glioma cells (**A**) Levels of extracellular glutamate were determined in three glioma cell lines, U87, LN229 and U118MG for 4 days. C8161 melanoma cells were used as positive control. Media only with no cells was used as negative control. (**B**) In parallel MTT assays were performed at the same time to be sure that the released glutamate measured was not due to lysed cells.

We next examined whether U87MG glioma cells secrete glutamate into the extracellular environment as we have demonstrated for GRM1^+^ melanoma cells. Indeed, glioma cells also release glutamate into the microenvironment (Figure 2). MTT assays were performed to ensure cell viability (Figure 2). We repeated the experiments in U118MG and LN229 cell lines, with very similar results (Figure 2). Thus, these results demonstrated the secretion of glutamate (GRM3’s natural ligand) by these GRM3^+^ glioma cells suggesting the potential for trophic autocrine/paracrine GRM3 stimulation in these glioma cells.

### Grm3 is functional as demonstrated by responsiveness to receptor agonist/antagonist

**Figure 3:**
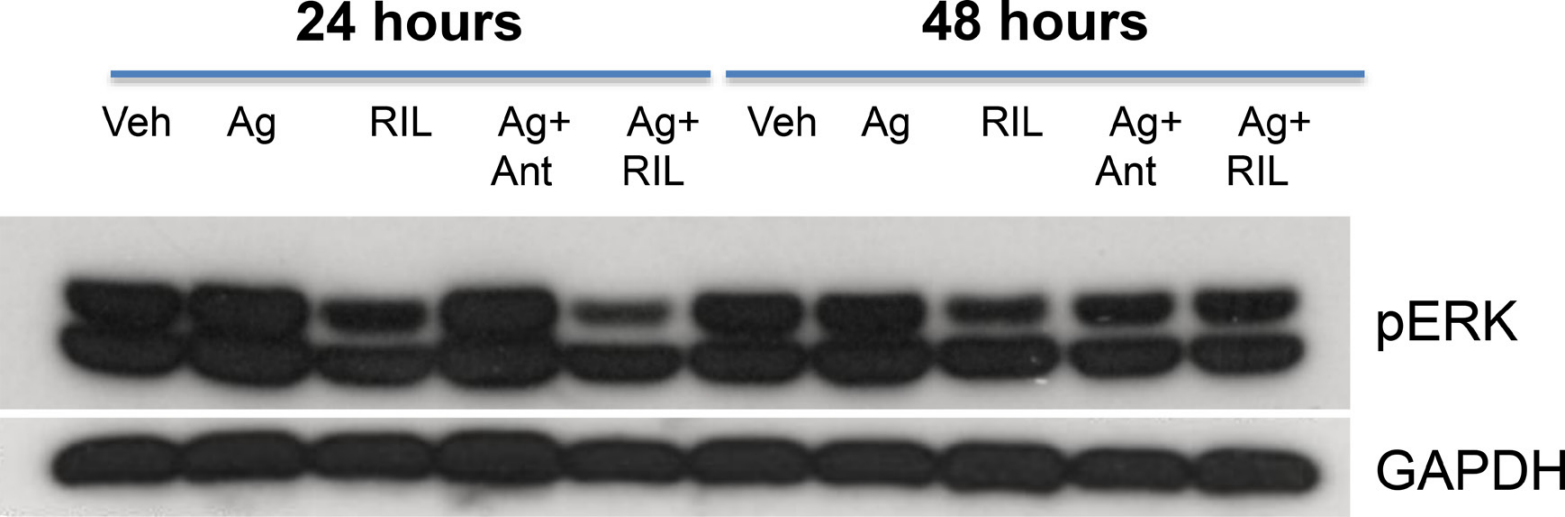
GRM3 in U87 cells is functional Western immunoblots of U87 cells treated with specific GRM3 agonist, LY379268 (Ag, 1 μM), riluzole (RIL, (1 μM), agonist plus GRM3 specific antagonist, LY341495 (Ant, (1 μM), or Ag plus RIL. Cells were pre-incubated with antagonist or RIL for 24 and 48 hours prior to agonist challenge. The membranes were probed with pERK antibody and GAPDH antibody as loading controls.

We wanted to assess whether GRM3 stimulation by receptor specific agonists in glioma cells could activate two well-known cell proliferation pathways, the MAPK and PI3/AKT pathways. Using a commercially available, specific GRM3 agonist (LY379268) we demonstrated increased phosphorylation of ERK but not AKT as a result of receptor stimulation. More importantly, this activation of ERK could be abrogated by pre-treatment with both a specific GRM3 antagonist (LY341495) and by riluzole (Figure 3).

### Riluzole promotes apoptotic glioma cell death *in vivo* and *in vitro*

**Figure 4:**
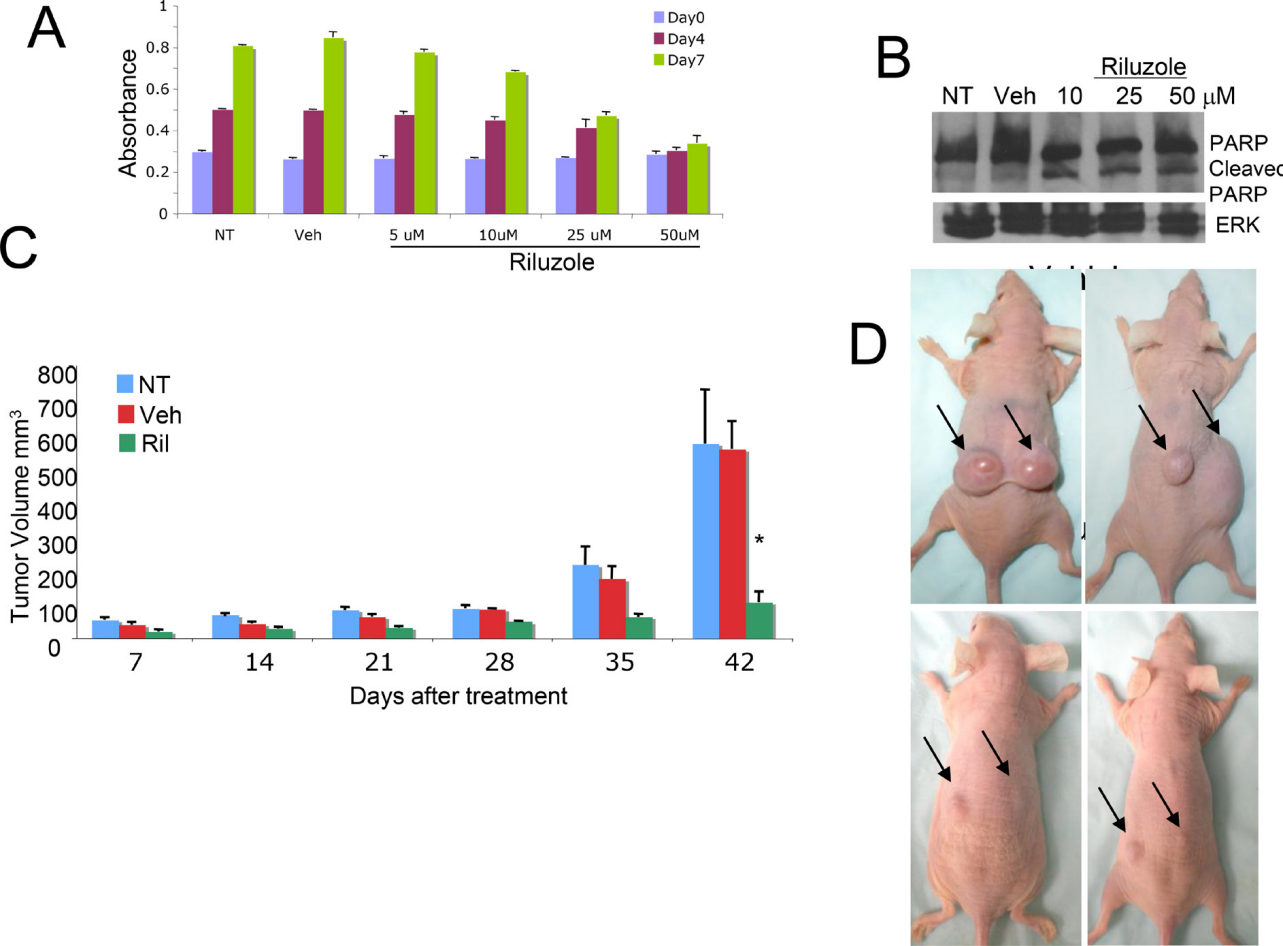
Cytotoxic effect of RIL on glioma cells (**A**) MTT cell viability/cell proliferation assays were performed with U87MG cells, the cells were either not treated (NT), treated with vehicle (Veh, DMSO) or riluzole at 5, 10, 25 or 50 μM for 7 days. (**B**) Protein lysates were prepared from U87MG glioma cells that were either not treated (NT), treated with vehicle (Veh, DMSO), or riluzole at 10, 25 or 50 μM for 4 days, and subjected to western immunoblots with antibody that recognizes both uncleaved and cleaved forms of PARP. (**C**) U87MG xenograft mice, 10^6^ cells were injected subcutaneously into the dorsal flanks of each mouse, when the tumor volumes reach about 100 mm^3^ the mice were divided into groups with similar tumor volume distribution and treatment is then initiated. The groups are no treatment NT), vehicle (Veh, DMSO) and riluzole (10 mg/kg), treatment was administrated via oral gavage daily for 42 days. ^*^p < 0.0001, comparison of riluzole treated mice with either no treatment or vehicle treated ones. (**D**) Top Panel: Vehicle treated mice or Bottom Panel: Riluzole treated mice at the end of the experiment.

We performed MTT assays on U87MG cells treated with vehicle or various concentrations of riluzole for 7 days; a dose-dependent decrease in the number of viable cells was detected (Figure 4A). In order to investigate if U87MG glioma cells also undergo apoptotic cell death as we have shown for riluzole-treated melanoma cells, we performed Western immunoblots using a well-known programmed cell death marker, the cleaved form of the Poly (ADP-ribose) polymerase, PARP. A dose-dependent increase in the cleaved PARP was detected in riluzole treated cell protein lysates (Figure 4B). Finally, a mouse xenograft study of no treatment (NT), vehicle (Veh) or riluzole (RIL) demonstrated a significant inhibition of tumor progression in U87MG-inoculated mice compared to no treatment or vehicle-treated ones (Figures 4C and 4D). We repeated the experiments in U118MG and LN229 cell lines, with similar results (Supplementary Figures 2 and 3).

### Riluzole treatment induces DNA damage in glioma cells

**Figure 5:**
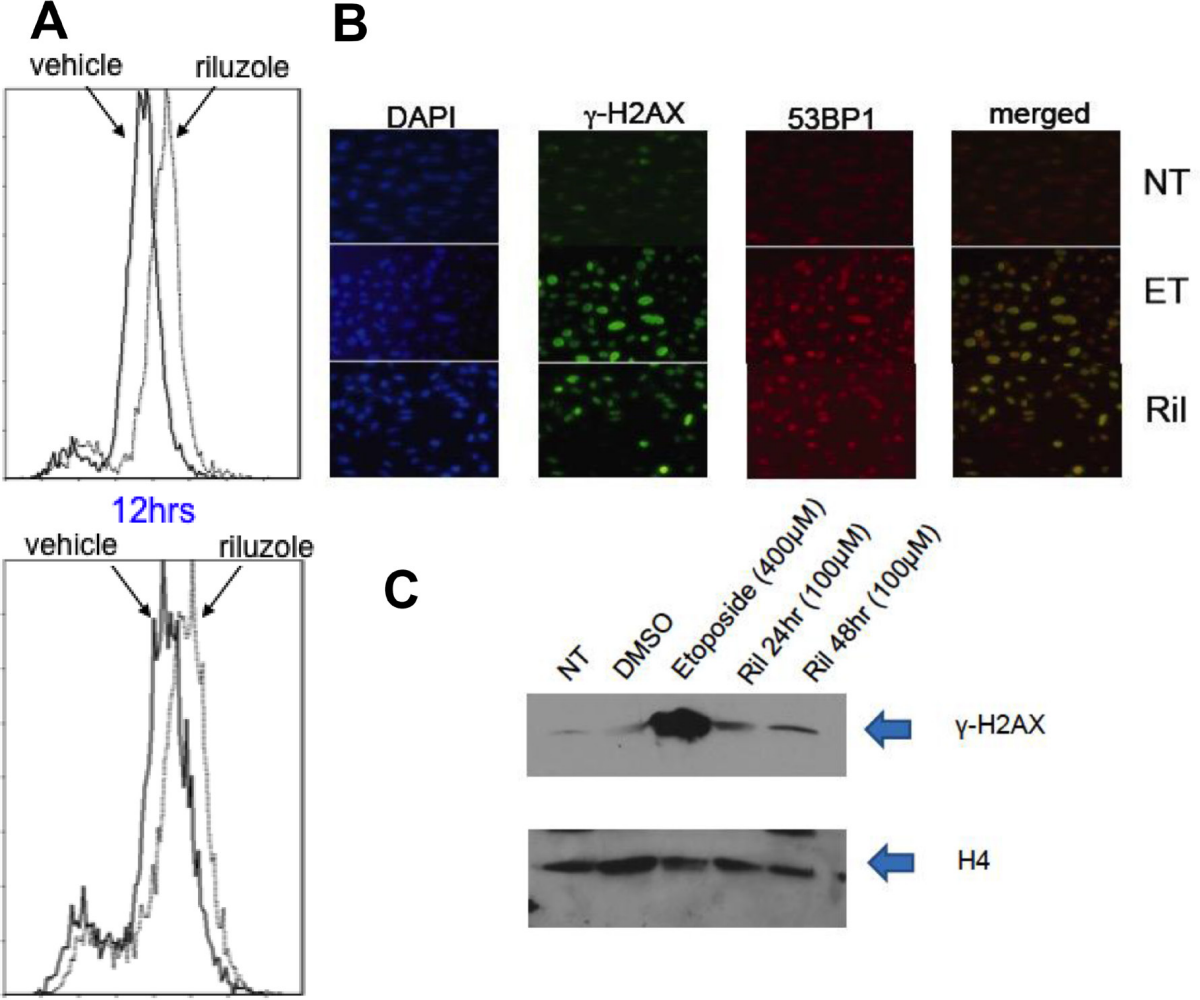
ROS mediated DNA damage in glioma cells treated with RIL (**A**) LN229 (top panel) and U87MG (bottom panel) cells were treated with vehicle (DMSO) or 10 uM riluzole for various time periods (12 hrs shown). The cells were incubated with 2′,7′-dichlorodihydrofluorescein diacetate (Sigma) for 20 min at 37° C. ROS-mediated oxidation of fluorochrome fluorescence was measured with flow cytometer, showing enhanced ROS with RIL. (**B**) Immunofluorescence stains for DAPI, γ-H2AX, 53BP1 or merged, showing co-localization after both etoposide (ET) and RIL in U87MG cells. (**C**) Western blot for γ-H2AX in cell lysates of U87MG cells treated with either no treatment (NT), vehicle (DMSO), etoposide (positive control) or riluzole. H4 is used as a loading control.

We hypothesized that riluzole may act through xCT, depleting glutathione levels and sensitizing cells to reactive oxygen species (ROS) [17]. If true, there would be important implications for concurrent treatment with radiotherapy, which relies on ROS-mediated double-strand DNA breaks. We evaluated levels of ROS in glioma cells treated with riluzole or vehicle and detected increased ROS (right shift of curve in Figure 5A). Furthermore, we observed co-localization of 53BP1 and γ-H2AX only in positive control etoposide or riluzole treated cells (Figure 5B), and elevated γ-H2AX levels in riluzole treated cells were further confirmed by Western blotting (Figure 5C).

### Riluzole enhances radiation-induced cytotoxicity in cells *in vitro*

**Figure 6:**
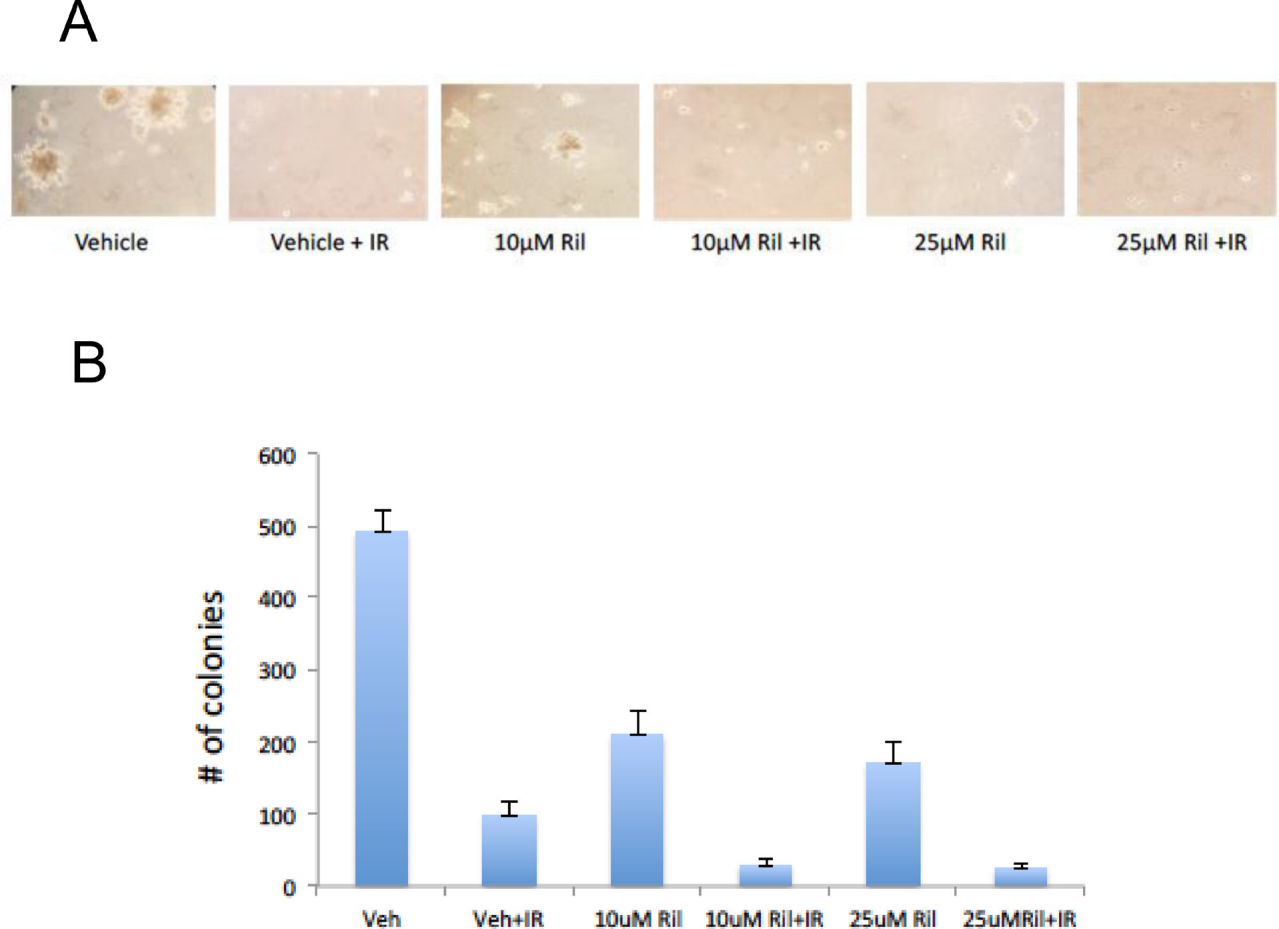
RIL + RT reduces anchorage-independent colony growth of glioma cells Three replicates of a soft agar colony assay were performed with U87MG cells in the presence of 10 μM riluzole, 25 μM riluzole, 10 μM riluzole with 12 Gy RT (delivered in 3 daily fractions), 25 μM riluzole with 12 Gy RT, vehicle solvent (DMSO) with 12 Gy RT, or vehicle solvent (DMSO) alone. Fresh media with 0.33% agarose with or without riluzole were placed on top of the cells once a week for three weeks. (**A**) Photomicrographs of three representative fields were taken after 3 weeks to count colonies formed in the agarose. The number of colonies greater than 30 mm were counted. (**B**) The number of colonies for each photomicrograph was determined using Image J and the sum totals were plotted relative to DMSO-treated cells, demonstrating fewer colonies with the combination of RIL and RT.

We examined the effects of concomitant riluzole and radiation in cultured cells in *in vitro* assays. Riluzole-pretreatment did not appear to enhance cell death in the classic clonogenic assays (data not shown). However, using a well-known anchorage-independent assay [18], riluzole enhanced radiation-induced cell cytotoxicity (Figure 6), suggesting paracrine effect.

## DISCUSSION

Our group has extensively studied and described the transforming ability of GRM1 in melanocytes, kidney epithelial cells and mammary epithelial cells *in vitro* and *in vivo* [19–21]. GRM1 is a member of the seven transmembrane G-protein coupled receptors (GPCRs), which have multiple synaptic functions. The natural ligand of GRM1 is L-glutamate; the CNS is a glutamate rich environment, thus making the study of glutamate signaling in CNS tumors an interesting and rational area for testing new hypotheses. In this report, we extended our insights in cellular glutamate signaling abnormalities through the related GRM3 receptor in the context of high-grade gliomas. Notably, we chose to examine GRM3 as other groups have demonstrated its significance. We too were able demonstrated that GRM3 is expressed in glioma cells, that these cells secrete glutamate, and that glutamate in turn activates cell surface GRM3 with resulting downstream activation of the MAPK pathway. We also demonstrated that interruption of this cascade with riluzole results in glioma cell cytotoxicity *in vivo* and *in vitro*, mediated through a mechanism of enhanced DNA damage. We currently do not know, but are studying, the precise mechanisms by which glioma cells secrete glutamate into the extracellular space, and the mechanisms by which RIL leads to DNA damage in this system. Our prior work in melanoma cells has implicated the cysteine-glutamate antiporter known as system X^c^ and indeed others have shown that this cell surface pump is instrumental in high-grade glioma pathogenesis [9, 22]. Cystine transport and supply is the rate-limiting step for cellular production of glutathione (GSH), a tripeptide of glutamate, cysteine (reduced cystine), and glycine. GSH is an important intracellular antioxidant that protects cells against damage from endogenous reactive oxygen species (ROS) and elevated GSH levels have been shown to confer radioresistance [23]. Chung and colleagues have demonstrated that pharmacologic inhibitors of system X^c^, a glutamate-cystine antiporter system composed of two proteins, xCT and CD98 [6], can completely deplete glioma cells of GSH with resulting tumor suppression [24]. This growth arrest can be reversed by rescue with a membrane-permeant GSH ester. This data has important implications as radiation-induced cell kill is dependent on ROS. We hypothesize that our observed finding of DNA damage with riluzole may be mediated by diminished glutathione and reduced reductive potential within the cell, a hypothesis that already has support in existing data reported by other as discussed above.

Excess glutamate in the microenvironment can lead to “excitotoxic death” of surrounding neurons, thus creating space for gliomas to expand. System X^c^ may also be the primary mechanism of glutamate release by glioma cells. Savaskan et al., have demonstrated that xCT levels are elevated in gliomas, xCT ablation by siRNA results in massive reduction in glutamate secretion, and that transferred, conditioned media from glioma cells with functional xCT can result in neuronal degeneration [9], but does not occur with media from cells with absent xCT.

As mentioned “excitotoxic” cell death has been recognized for decades [2] as the final common death pathway for many neurological injuries [3, 4]. Recent data has also demonstrated that this excess glutamate from glioma cells can contribute to glioma-associated seizures, a major cause of morbidity for patients. Buckingham and colleagues demonstrated, in an intracranial glioma model, that glutamate-release from the xenografted tumors correlates with epileptiform excitability, and that this could be abrogated by blocking glutamate-release using sulfasalazine [25]. Sulfasalazine, an orally available and well-tolerated salicylate used for inflammatory bowel disease, is a specific inhibitor of system X^c^. Moreover, glutamate autocrine and paracrine stimulation of cell surface metabotropic receptors may facilitate glioma growth and motility. Arcella et al., assessed whether GRM3 stimulation by receptor specific agonists could activate two well-known cell proliferation pathways, the MAPK and PI3/AKT pathways in U87 cells [26]. Using a commercially available, specific GRM3 agonist (LY379268) they demonstrated increased phosphorylation of ERK and AKT as a result of receptor stimulation. More importantly, this activation of ERK and AKT was abrogated by a specific GRM3 antagonist (LY341495). In summary, it appears that decreased uptake and excess secretion of extracellular glutamate in the microenvironment facilitates intracranial glioma progression and expansion by several mechanisms.

Glutamate abnormalities are now known to be involved in various tissue types and various human malignancies. A phase II trial of a non-competitive glutamate inhibitor with temozolamide and irradiation has been reported in glioblastoma [27]. This was a negative trial, but targeted ionotropic glutamate receptors with talampanel. In contrast, most of the existing data supports a role for type I/II metabotropic receptors (GRM1 and GRM3) and the system X^c^ antiporter. Riluzole, a glutamate release inhibitor, is an oral agent with CNS penetration that has been used chronically in patients with neurodegenerative disorders and blocks glutamate release.

Indeed other groups have very recently demonstrated that riluzole specifically inhibits cancer cells across a spectrum of tumor types. Lemieszek et al demonstrated G2/M arrest with riluzole as a potential mechanism of cell death and anti-migrative in a number of cancer cell lines [28]. Zhang et al demonstrated reduced viability, invasion and migration of U87 cells treated with riluzole [29]. In contrast to our report, they demonstrated mediation of riluzole’s effects through AKT-mTOR signaling, whereas our findings implicate MAPK signaling. In a significant corroboration of our report, Yohay et al demonstrated that riluzole delivered either locally (loaded on a polymer) or systemically had significant anti-glioma effects, and strikingly the effect of the of systemic administration synergized strongly with radiotherapy [30]. Others, too, have demonstrated the activity of riluzole or LY341495 on proliferation, survival, and/or migration of glioma cells [31, 32].

Taken together, our work as well as that of others leads us to our current working hypothesis in which overexpression of system X^c^ in gliomas increases intracellular glutathione (protecting cells from ROS-related stresses) and increases extracellular glutamate which then acts as a paracrine/autocrine trophic factor for glioma progression through stimulation of GRM3. Interruption of this glutamate release by RIL abrogates trophic signaling through the MAPK pathway and reduces intracellular glutathione, thus increasing cellular vulnerability to ROS-related DNA damage. Our current work is aimed at confirming this model and identifying the mechanisms by which RIL acts on glutamate release. Given the availability of RIL and other glutamate blockers, our work has significant translational implications.

## MATERIALS AND METHODS

### Commercial cell lines

U87MG, T98G, LN229 and U118MG were obtained from ATCC. Cells were cultured in monolayer at 37° C in a 5% CO2 humidified incubator, in RPMI (InVitrogen) supplemented with 10% fetal bovine serum (Sigma).

### Culture of human glioma cells

De-identified primary human tumor samples were obtained from patients undergoing craniotomy and resection at Robert Wood Johnson Medical School – Rutgers University under an IRB approved protocol. Cells were obtained through mechanical dissociation of the tumor tissue using a blade and plated in DMEM/F12 medium in the presence of B-27 Supplement, 20 ng/ml of human recombinant EGF and human recombinant FGF. The following day, the culture was collected, incubated with Accutase at 37° C and passed through a needle to obtain a single cell suspension and re-plated in the same supplemented medium. Upon reaching confluency, half of the neurospheres were plated into EMEM with serum to create an adherent monolayer population.

### MTT cell viability assay

Cells were seeded in 96 well plates and exposed to drug and radiation the following day for 5 consecutive days. 48 hours later, 3-(4,5-dimethylthiazol**-**2-yl)-2,5-diphenyltetrazolium bromide (MTT) (Sigma) was added to wells and incubated at 37° C; crystals were dissolved in DMSO and read in a fluorescent plate reader at 570nm.

### Western immunoblots

Western immunoblots of U87MG hGRM1-positive human glioma cells either treated with riluzole for 24 hours or no treatment were conducted. Both sets of cells were irradiated at 2 or 4 Gy. Lysates were made at 24, 48 or 72 hours after irradiation. Protein lysates were prepared by washing cells with PBS, adding extraction buffer (50 mM Tris, 150 mM NaCl, 1mM EDTA, pH 8.0, 1% NP40, 5% glycerol, 1mM Dithiothreitol, complete protease inhibitor cocktail (Roche) and phosphatase inhibitors I and II (Sigma). The samples were resolved in 10% SDS-PAGE gels (Bio-Rad), after which they were transferred onto nitrocellulose membranes. Membranes were blocked with 5% milk and 1% bovine serum albumin and then probed with antibodies against PARP, cleaved caspase-3, or γH2AX. The same blot was probed with α-tubulin or loading controls to show equal loading.

### Immunofluorescence

Chamber slides were coated with poly-ornithine for 1 hour at 37° C, rinsed in water and subsequently coated with laminin for 1 hour at 37° C, followed by a rinse with water. Cells growing in neurospheres media were plated and allowed to attach overnight before 5-day treatment scheme. At the specified time point, immunofluorescence staining was performed; briefly: cells were fixed in Formalde - Fresh (Fischer Scientific), washed and pemeabilized in a 0.2% Triton-X-100 PBS solution for 5 minutes. An hour blocking period followed in a PBS solution containing 4% BSA, 1% FBS for 1 hour at room temperature. Primary antibodies were incubated for 1 hour at room temperature in blocking solution followed by incubation with secondary antibodies for 1 hour at room temperature. Slides were mounted with Hard Set Mounting Media with DAPI (VectorShield) before imaging with a fluorescent microscope. We used the antibody to γH2AX (Millipore) to visualize cells with DNA damage. Immediately after double strand breakage (DSB), γH2AX forms bright nuclear foci on immunofluorescent microscopy. The presence of γH2AX foci is widely regarded as a surrogate for and a sensitive marker of DSB in cells [33–36] and have been shown to be specific for DSB [37].

### ROS experiment

Cells are seeded at 3 × 10^5^ per well in a 6 well plate and treated as indicated. After treatment cells are loaded with dihydrorhodamine 123 (DHR123) for at least 20 min at 37° C in a humidified 5% CO2/95% air incubator. The plates are washed by several changes of growth medium, trypsinized and cells collected by centrifugation at 300 × g, washed, and resuspended in PBS. ROS levels are immediately measured by the Flow Cytometry Facility Core at Rutgers University using a Beckman Coulter FC500 Analyser (Epics XL-MCL model). Rhodamine 123 derived from DHR123 by oxidation is excited with an air-cooled argon ion laser at 488 nm. Fluorescent emission of the marker is detected between 525 and 535 nm. Cell debris and multi-cell aggregates are electronically gated out.

### Murine xenograft model

One million hGRM-positive U87MG cells were injected into the flanks of 6 weeks old nude mice (Taconic). When the tumors reached approximately 60–100 mm^3^ the mice were randomly divided into 3 groups of 10 mice each: Group 1 was left untreated (No Treatment or NT), group 2 was treated with the DMSO vehicle only (Vehicle), and group 3 was treated with 10mg/kg of riluzole by oral gavage for 42 days (riluzole). We monitored tumor progression in each condition and measuring the tumors with vernier calipers twice per week. The experiment was terminated for tumor burden. The animals were sacrificed, and half of the samples were snap-frozen for further molecular studies and the other half was dropped in formalin for histological analysis.

### Soft agar colony formation assay

One hundred thousand cells were plated in 0.33% agarose on top of a 0.5% agarose bed with the appropriate concentration of riluzole. At 24 hours, the designated plates of cells were exposed to 4 Gy of gamma irradiation, which was then repeated at 24 hour intervals for a total of three consecutive days. Fresh media with 0.33% agarose with or without riluzole were placed on top of the cells once a week for three weeks. The number of colonies greater than 30 mm were counted. The Keyence BZ-X710 microscope was utilized to obtain photographs of the colonies formed in the agarose.

### Statistics

Significance was determined using Student’s two-tailed *t*-test. A *p* value of < 0.05 was considered significant.

## SUPPLEMENTARY MATERIALS FIGURES



## Abbreviations

BSA: bovine serum albumin
CNS: central nervous system
DSB: double-strand break
GRM1: metabotropic glutamate receptor 1
GRM3 and mGluR3: metabotropic glutamate receptor 3
IR: Ionizing radiation
MGMT: O^6^-methylguanine-DNA methyltransferase
RIL: riluzole
PARP: Poly (ADP-ribose) polymerase
ROS: reactive oxygen species
RT: radiation therapy

## References

[1] Stupp R, Hegi ME, Mason WP, van den Bent MJ, Taphoorn MJ, Janzer RC, Ludwin SK, Allgeier A, Fisher B, Belanger K, Hau P, Brandes AA, Gijtenbeek J (2009). Effects of radiotherapy with concomitant and adjuvant temozolomide versus radiotherapy alone on survival in glioblastoma in a randomised phase III study: 5-year analysis of the EORTC-NCIC trial. Lancet Oncol.

[2] Olney JW, Ho OL, Rhee V (1971). Cytotoxic effects of acidic and sulphur containing amino acids on the infant mouse central nervous system. Exp Brain Res.

[3] Lipton SA, Rosenberg PA (1994). Excitatory amino acids as a final common pathway for neurologic disorders. N Engl J Med.

[4] Choi DW (1988). Glutamate neurotoxicity and diseases of the nervous system. Neuron.

[5] Danbolt NC (2001). Glutamate uptake. Prog Neurobiol.

[6] Ye ZC, Rothstein JD, Sontheimer H (1999). Compromised glutamate transport in human glioma cells: reduction-mislocalization of sodium-dependent glutamate transporters and enhanced activity of cystine-glutamate exchange. J Neurosci.

[7] de Groot JF, Liu TJ, Fuller G, Yung WK (2005). The excitatory amino acid transporter-2 induces apoptosis and decreases glioma growth *in vitro* and *in vivo*. Cancer Res.

[8] Sontheimer H (2008). A role for glutamate in growth and invasion of primary brain tumors. J Neurochem.

[9] Savaskan NE, Heckel A, Hahnen E, Engelhorn T, Doerfler A, Ganslandt O, Nimsky C, Buchfelder M, Eyupoglu IY (2008). Small interfering RNA-mediated xCT silencing in gliomas inhibits neurodegeneration and alleviates brain edema. Nat Med.

[10] Ciceroni C, Arcella A, Mosillo P, Battaglia G, Mastrantoni E, Oliva MA, Carpinelli G, Santoro F, Sale P, Ricci-Vitiani L, De Maria R, Pallini R, Giangaspero F (2008). Type-3 metabotropic glutamate receptors negatively modulate bone morphogenetic protein receptor signaling and support the tumourigenic potential of glioma-initiating cells. Neuropharmacology.

[11] Marin YE, Namkoong J, Cohen-Solal K, Shin SS, Martino JJ, Oka M, Chen S (2006). Stimulation of oncogenic metabotropic glutamate receptor 1 in melanoma cells activates ERK1/2 via PKCepsilon. Cell Signal.

[12] Namkoong J, Shin SS, Lee HJ, Marin YE, Wall BA, Goydos JS, Chen S (2007). Metabotropic glutamate receptor 1 and glutamate signaling in human melanoma. Cancer Res.

[13] Pollock PM, Cohen-Solal K, Sood R, Namkoong J, Martino JJ, Koganti A, Zhu H, Robbins C, Makalowska I, Shin SS, Marin Y, Roberts KG, Yudt LM (2003). Melanoma mouse model implicates metabotropic glutamate signaling in melanocytic neoplasia. Nat Genet.

[14] Doble A (1996). The pharmacology and mechanism of action of riluzole. Neurology.

[15] McGeer EG, McGeer PL (2005). Pharmacologic approaches to the treatment of amyotrophic lateral sclerosis. BioDrugs.

[16] Miller R (2003). Riluzole for ALS: what is the evidence?. Amyotroph Lateral Scler Other Motor Neuron Disord.

[17] Wall BA, Wangari-Talbot J, Shin SS, Schiff D, Sierra J, Yu LJ, Khan A, Haffty B, Goydos JS, Chen S (2014). Disruption of GRM1-mediated signalling using riluzole results in DNA damage in melanoma cells. Pigment Cell Melanoma Res.

[18] Le MN, Chan JL, Rosenberg SA, Nabatian AS, Merrigan KT, Cohen-Solal KA, Goydos JS (2010). The glutamate release inhibitor Riluzole decreases migration, invasion, and proliferation of melanoma cells. J Invest Dermatol.

[19] Shin SS, Namkoong J, Wall BA, Gleason R, Lee HJ, Chen S (2008). Oncogenic activities of metabotropic glutamate receptor 1 (Grm1) in melanocyte transformation. Pigment Cell Melanoma Res.

[20] Zhu H, Reuhl K, Zhang X, Botha R, Ryan K, Wei J, Chen S (1998). Development of heritable melanoma in transgenic mice. J Invest Dermatol.

[21] Zhu H, Ryan K, Chen S (1999). Cloning of novel splice variants of mouse mGluR1. Brain Res Mol Brain Res.

[22] Chen RS, Song YM, Zhou ZY, Tong T, Li Y, Fu M, Guo XL, Dong LJ, He X, Qiao HX, Zhan QM, Li W (2009). Disruption of xCT inhibits cancer cell metastasis via the caveolin-1/beta-catenin pathway. Oncogene.

[23] Mitchell JB, Cook JA, DeGraff W, Glatstein E, Russo A (1989). Glutathione modulation in cancer treatment: will it work?. Int J Radiat Oncol Biol Phys.

[24] Chung WJ, Lyons SA, Nelson GM, Hamza H, Gladson CL, Gillespie GY, Sontheimer H (2005). Inhibition of cystine uptake disrupts the growth of primary brain tumors. J Neurosci.

[25] Buckingham SC, Campbell SL, Haas BR, Montana V, Robel S, Ogunrinu T, Sontheimer H (2011). Glutamate release by primary brain tumors induces epileptic activity. Nat Med.

[26] Arcella A, Carpinelli G, Battaglia G, D'Onofrio M, Santoro F, Ngomba RT, Bruno V, Casolini P, Giangaspero F, Nicoletti F (2005). Pharmacological blockade of group II metabotropic glutamate receptors reduces the growth of glioma cells *in vivo*. Neuro Oncol.

[27] Grossman SA, Ye X, Chamberlain M, Mikkelsen T, Batchelor T, Desideri S, Piantadosi S, Fisher J, Fine HA (2009). Talampanel with standard radiation and temozolomide in patients with newly diagnosed glioblastoma: a multicenter phase II trial. J Clin Oncol.

[28] Lemieszek MK, Stepulak A, Sawa-Wejksza K, Czerwonka A, Ikonomidou C, Rzeski W (2018). Riluzole Inhibits Proliferation, Migration and Cell Cycle Progression and Induces Apoptosis in Tumor Cells of Various Origins. Anticancer Agents Med Chem.

[29] Zhang C, Yuan XR, Li HY, Zhao ZJ, Liao YW, Wang XY, Su J, Sang SS, Liu Q (2015). Anti-cancer effect of metabotropic glutamate receptor 1 inhibition in human glioma U87 cells: involvement of PI3K/Akt/mTOR pathway. Cell Physiol Biochem.

[30] Yohay K, Tyler B, Weaver KD, Pardo AC, Gincel D, Blakeley J, Brem H, Rothstein JD (2014). Efficacy of local polymer-based and systemic delivery of the anti-glutamatergic agents riluzole and memantine in rat glioma models. J Neurosurg.

[31] Tsuchioka M, Hisaoka K, Yano R, Shibasaki C, Kajiatani N, Takebayashi M (2011). Riluzole-induced glial cell line-derived neurotrophic factor production is regulated through fibroblast growth factor receptor signaling in rat C6 glioma cells. Brain Res.

[32] Yelskaya Z, Carrillo V, Dubisz E, Gulzar H, Morgan D, Mahajan SS (2013). Synergistic inhibition of survival, proliferation, and migration of U87 cells with a combination of LY341495 and Iressa. PloS One.

[33] Fillingham J, Keogh M, Krogan N (2006). Gamma H2AX and its role in DNA double-strand break repair Auger electrons—a nanoprobe for structural, molecular, and cellular processes. Biochem Cell Biol.

[34] Pilch D, Sedelnikova O, Redon C, Celeste A, Nussenzweig A, Bonner W (2003). Characteristics of gamma-H2Ax foci at DNA double-strand breaks sites. Biochem Cell Biol.

[35] Rogakou E, Pilch D, Orr A, Ivanova V, Bonner W (1998). DNA double-strand breaks induce histone H2AX phosphorylation on serine 139. J Biol Chem.

[36] Sedelnikova O, Rogakou E, Panyutin I, Bonner W (2002). Quantitative detection of (125)IdU-induced DNA double-strand breaks with gamma-H2AX antibody. Radiat Res.

[37] Takahashi A, Ohnishi T (2005). Does Gamma H2AX foci formation depend on the presence of DNA double strand breaks?. Cancer Lett.

