# Supplementary material for: The glutamate release inhibitor riluzole increases DNA damage and enhances cytotoxicity in human glioma cells, in vitro and in vivo
Source: Oncotarget. 2019 Apr 19;10(29):2824–34. doi: 10.18632/oncotarget.26854 (PMC6497458; doi:10.18632/oncotarget.26854)
Supplement: Supplementary file 1 [file oncotarget-10-2824-s001.pdf]

The glutamate release inhibitor riluzole increases DNA damage and enhances cytotoxicity in human glioma cells, *in vitro* and *in vivo*

SUPPLEMENTARY MATERIALS

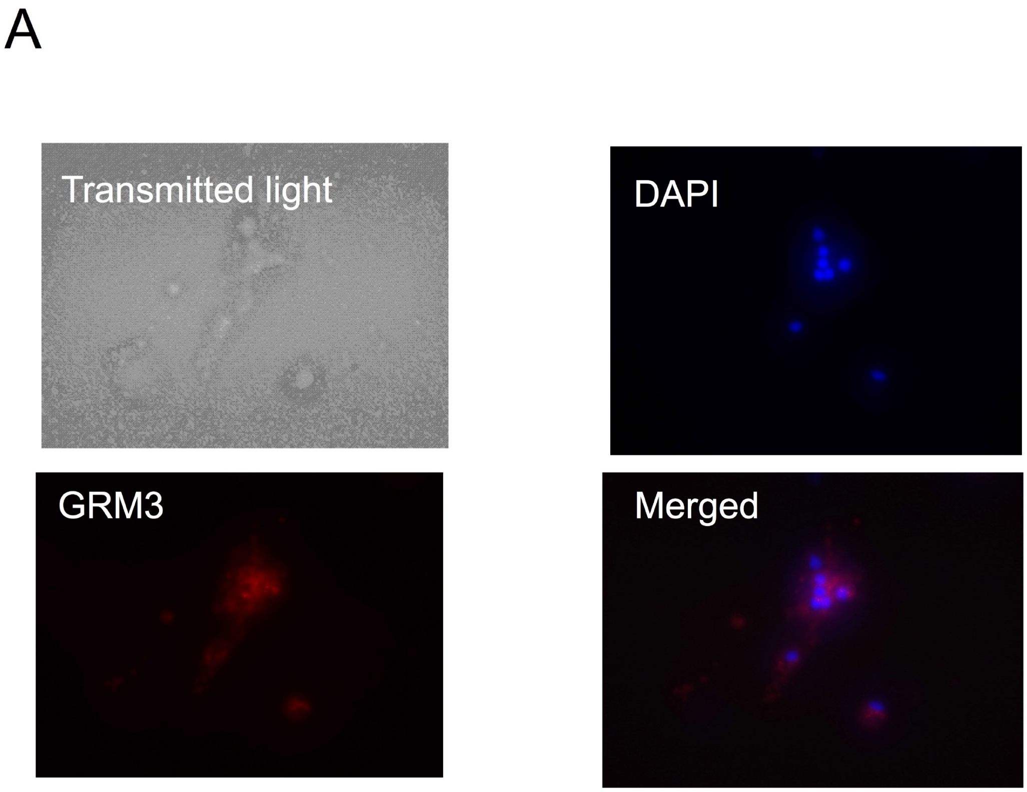

Supplementary Figure 1: GRM3 is expressed in human gliomas. Immunofluorescence demonstration of DAPI, rhodamine-GRM3 and merged of GRM3 and DAPI in an example of primary patient derived cells.

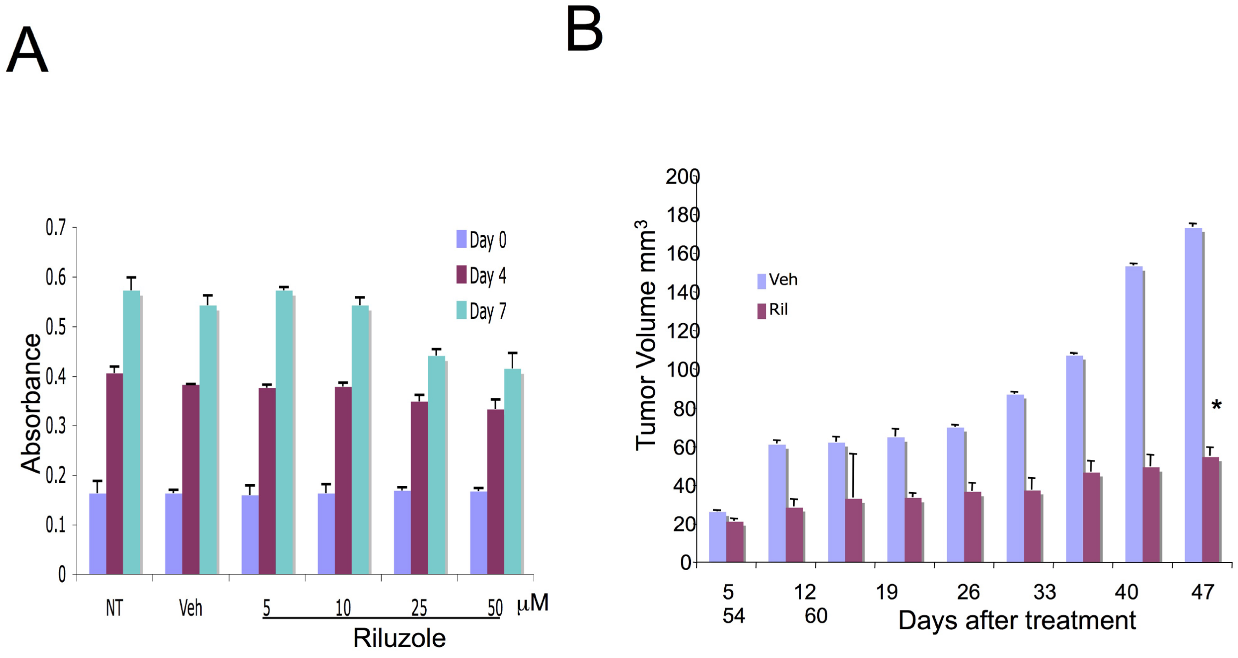

Supplementary Figure 2: Cytotoxic effect of RIL on glioma cells. (**A**) MTT cell viability/cell proliferation assays were performed with U118MG cells, the cells were either not treated (NT), treated with vehicle (Veh, DMSO) or riluzole at 5, 10, 25 or 50 mM for 7 days. (**B**) U118MG xenograft mice, 10^6 cells were injected subcutaneously into the dorsal flanks of each mouse, when the tumor volumes reach about 10 mm^3 the mice were divided into groups with similar tumor volume distribution and treatment is then initiated. The groups are no treatment (NT), vehicle (Veh, DMSO) and riluzole (10 mg/kg), treatment was administrated via oral gavage daily for 42 days. $^*p < 0.05$, comparison of riluzole treated mice with either no treatment or vehicle treated ones.

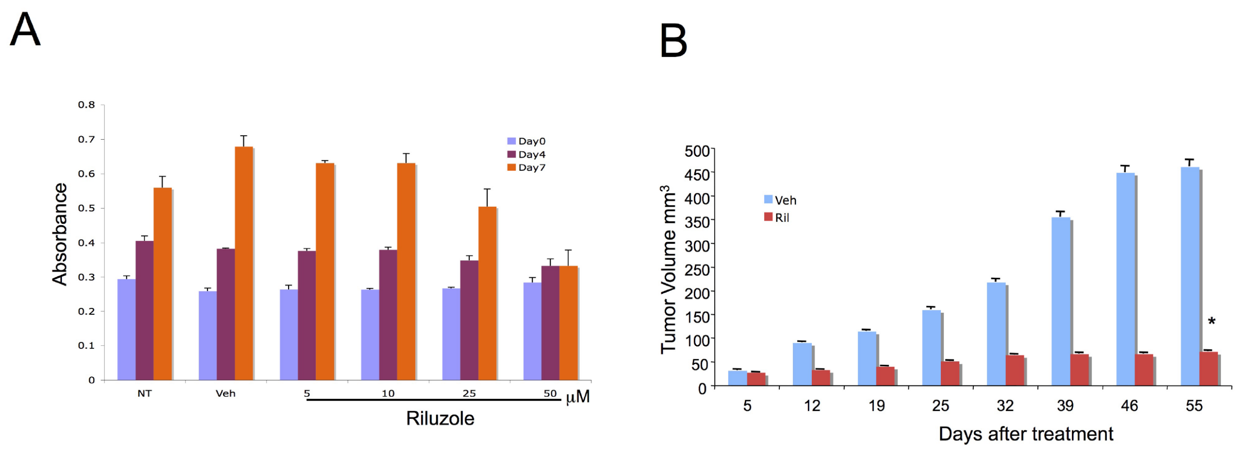

Supplementary Figure 3: Cytotoxic effect of RIL on glioma cells. (**A**) MTT cell viability/cell proliferation assays were performed with LN229 cells, the cells were either not treated (NT), treated with vehicle (Veh, DMSO) or riluzole at 5, 10, 25 or 50 mM for 7 days. (**B**) LN229 xenograft mice, 10^6 cells were injected subcutaneously into the dorsal flanks of each mouse, when the tumor volumes reach 6–10 mm^3 the mice were divided into groups with similar tumor volume distribution and treatment is then initiated. The groups are no treatment (NT), vehicle (Veh, DMSO) and riluzole (10 mg/kg), treatment was administrated via oral gavage daily for 42 days. $^*p < 0.0001$, comparison of riluzole treated mice with either no treatment or vehicle treated ones.
